# GPCR-GO: Relation-aware graph learning for predicting Gene Ontology terms of G protein-coupled receptors

**DOI:** 10.1371/journal.pcbi.1014718

**Published:** 2026-08-28

**Authors:** Anchi Sun, Yongjing Hao, Yijie Ding, Jing Chen, Hongjie Wu

**Affiliations:** 1 School of Electronic and Information Engineering, Suzhou University of Science and Technology, Suzhou, Jiangsu, China; 2 Jiangsu Province Key Laboratory of Intelligent Building Energy Efficiency, Suzhou University of Science and Technology, Suzhou, Jiangsu, China; Ocean University of China, CHINA

## Abstract

G protein-coupled receptors (GPCRs) are central membrane receptors and major therapeutic targets. However, predicting GPCR function remains difficult because experimentally annotated receptors are scarce, functional labels follow a long-tailed distribution, and structural information remains underused. Here, we present GPCR-GO, a relation-aware heterogeneous graph attention framework that integrates structural similarity with protein-protein interactions (PPIs). GPCR-GO uses the Dictionary of Protein Secondary Structure (DSSP) to transform three-dimensional protein structures into residue-level structural descriptors, aggregates these descriptors into protein-level structural vectors, and uses the resulting vectors to define structural-similarity edges. The framework builds a heterogeneous graph linking proteins and Gene Ontology (GO) terms through PPI edges, structural-similarity edges, GO hierarchy edges, and reviewed protein–GO annotations. Relation-aware graph attention aggregates complementary biological signals, whereas graph decomposition, hard negative mining, and semi-supervised learning improve learning under sparse supervision and class imbalance. On the held-out GPCR test split, GPCR-GO outperforms existing methods and achieves F-score (Fmax) values of 0.514, 0.767, and 0.631 on biological process (BP), cellular component (CC), and molecular function (MF), respectively. These results show that structure-derived relations complement curated annotation and support accurate GPCR function prediction under limited supervision.

## Author summary

G protein-coupled receptors are important drug targets, but many members of this family still lack reliable functional annotations. This gap matters because experimental annotation is slow and existing computational predictors often struggle when labels are rare, proteins have limited homology, or structural signals are only weakly used. We developed GPCR-GO to address this problem by combining several sources of biological evidence in a single graph-learning framework. In our model, GPCR proteins, Gene Ontology terms, protein-protein interactions, ontology relations, curated annotations, and structure-derived similarity links are represented together. We also use semi-supervised learning, hard negative mining, and graph decomposition to improve learning when annotated GPCR examples are limited. Across biological process, cellular component, and molecular function tasks, GPCR-GO performs better than several sequence-, structure-, ontology-, and graph-based baselines on the held-out GPCR test split. These results suggest that structure-derived relationships can complement curated interactions and annotations, supporting more accurate function prediction for GPCRs under sparse supervision.

## Introduction

Proteins drive virtually every biological process, and reliable functional annotation is central to understanding cellular systems, disease mechanisms, and therapeutic opportunity [1]. Gene Ontology (GO) organizes protein function into biological process (BP), cellular component (CC), and molecular function (MF), making protein function prediction an intrinsically multi-label, hierarchy-aware problem [2,3]. High-throughput sequencing has generated protein sequences at a pace far exceeding experimental annotation, leaving only a small fraction of proteins supported by curated GO evidence [1,4]. Because protein function depends on sequence, structure, interaction networks, and cellular context, accurate prediction increasingly depends on integrating heterogeneous biological information [5–7].

G protein-coupled receptors (GPCRs) are among the largest membrane-protein families and account for roughly one-third of all therapeutic targets [8]. They are central to sensory perception, immune signaling, endocrine regulation, and nervous-system activity, which makes them especially important in drug discovery and precision medicine [8,9]. Yet GPCRs are difficult to annotate: their architectures are complex, their transmembrane regions are highly conserved, and functional diversity is often hard to infer from sequence alone, especially for newly identified receptors with unknown roles [8]. In the GPCR collection compiled for this study, only 3,465 UniProt entries were reviewed, a scale that limits fully supervised family-specific modeling. Predictive frameworks that integrate structure, interaction, and other complementary modalities are therefore needed for systematic GPCR annotation [5–7]. Related biomedical prediction studies have shown that multimodal graph attention and graph-transformer architectures can integrate complementary drug, target, disease, and interaction information for drug-target affinity prediction, drug-disease association prediction, and drug repositioning [10–12].

Previous studies have integrated sequence, structure, and interaction data to improve protein function prediction, but GPCR modeling remains constrained by limited data and incomplete use of structural information. Early deep-learning methods, including DeepGO [13] and DeepGOPlus [14], relied mainly on sequence-derived signals and were therefore limited for low-homology proteins. Later topology-aware approaches, such as PANDA2 [15] and DeepGraphGO [16], incorporated structural domains, conserved motifs, and protein-protein interaction (PPI) networks. Advances in structure prediction—most notably AlphaFold [17]—have since enabled structure-aware predictors such as Struct2GO [18] and statistics-informed graph networks [19]. Even so, many structure-driven models remain highly sensitive to input quality and derived features, often by simply concatenating structural descriptors with sequence features. This can introduce redundancy, obscure long-range dependencies, and weaken the identification of functionally important residues [20]. In parallel, self-supervised protein language models such as ProtTrans [21] and ESM [22] have established a strong pretrain–fine-tune paradigm for low-annotation settings. Yet functional databases remain incomplete and biased, supervision remains sparse, and multifunctionality and context dependence continue to complicate prediction in families such as GPCRs [4].

Motivated by these limitations, we propose a heterogeneous graph framework that integrates structural information, PPIs, and functional ontology relations for GPCR function prediction. The framework is designed to (i) alleviate the scarcity of labeled GPCR samples through semi-supervised learning [23], and (ii) supplement conventional PPI networks with high-confidence structural-similarity edges that provide an additional, structurally grounded source of protein relatedness.

The main contributions are fourfold. First, we use the Dictionary of Protein Secondary Structure (DSSP) [24] to derive residue-level secondary-structure and local-environment features from three-dimensional protein structures and use them to define structural-similarity edges. Second, we jointly model PPI and structural-similarity edges within a heterogeneous graph neural network to capture complementary semantics across relation types [11,12,25]. Third, we combine hard negative mining with semi-supervised learning on large-scale unlabeled data to improve robustness under sparse supervision. Fourth, we introduce a multi-relation-enhanced graph decomposition network [26] with regularized representation learning to strengthen heterogeneous information modeling and downstream classification.

## Results

### Overview of the GPCR-GO framework

**Fig 1:**
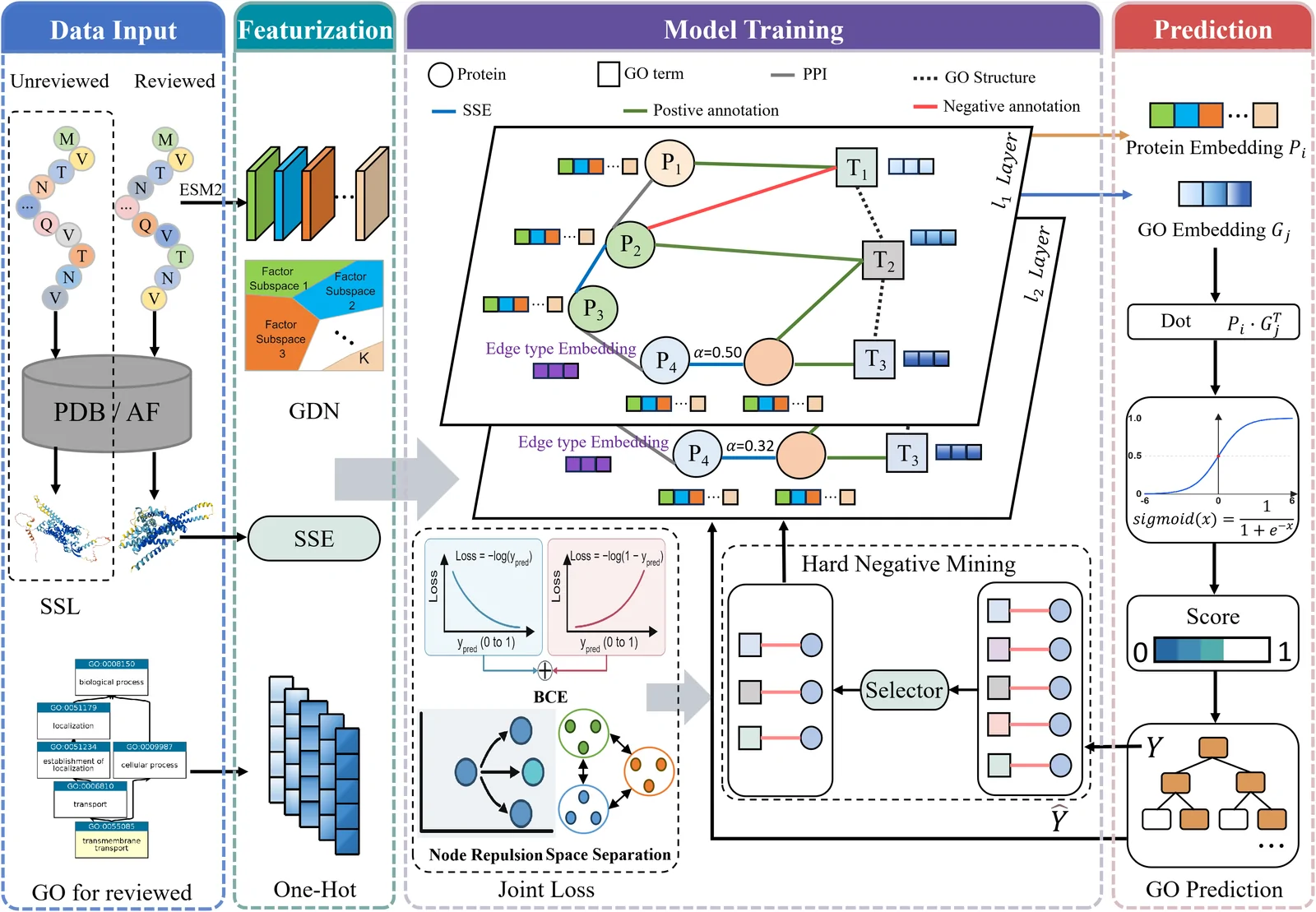
Architecture of GPCR-GO. Data input follows a semi-supervised setting in which reviewed and unreviewed proteins share the same sequence and structure processing pipeline. Both provide protein sequences and three-dimensional structures (PDB/AF); only reviewed proteins provide GO annotations, whereas unreviewed proteins enter the model as unlabeled nodes for semi-supervised learning. ESM2 encodes protein sequences, GDN decomposes protein representations into factor subspaces, DSSP-derived secondary-structure and solvent-accessibility features are extracted from three-dimensional structures, and GO term nodes are one-hot encoded. The heterogeneous graph contains protein nodes, GO nodes, PPI edges, GO hierarchical edges, structural-similarity edges, and observed positive protein–GO annotation edges from reviewed proteins. Multi-layer graph attention and hard negative mining are used during training. The loss combines binary cross-entropy with node-repulsion and space-separation regularizers. Functional association scores are obtained by dot products between protein and GO embeddings followed by a sigmoid transformation.

The overall framework of GPCR-GO is shown in Fig 1. Under the semi-supervised training scheme, reviewed and unreviewed proteins pass through the same sequence and structure processing pipeline and are incorporated as protein nodes in the heterogeneous graph. Reviewed proteins provide GO annotations, whereas unreviewed proteins remain unlabeled and contribute through representation learning and message passing. Protein nodes and GO term nodes are embedded in a unified heterogeneous graph, whose biological edges comprise PPIs, GO ontology relations, structural-similarity relations, and observed positive protein–GO annotations from reviewed proteins. Each node type is initialized with its corresponding attributes—protein sequence features or GO term one-hot encodings—and multi-layer heterogeneous graph attention is then used for deep feature aggregation across relations. Finally, a decoder scores protein–GO pairs to perform multi-label function prediction. This design integrates multimodal biological evidence while respecting the GO hierarchy and the reviewed-only supervision scheme, thereby improving generalization and predictive performance. A concise mapping of each information source to its graph component, input representation, role in the model, and prediction target is provided in Table E in S1 Appendix.

In this study, reviewed protein–GO pairs lacking current positive evidence were used as the candidate negative pool, and sampled pairs from this pool are referred to as *unannotated negatives*: training negatives defined by the current annotation snapshot. For readability, after this definition we use negative samples to denote these sampled unannotated negatives.

Under the fixed 8:1:1 data split used throughout this study, we summarized the reviewed GPCR subset to characterize label coverage, annotation density, and GO-term specificity. We also evaluated the direct transferability of released general protein-function predictors before GPCR-specific retraining, using the same held-out test split and matched evaluation protocol.

### Dataset statistics and challenge analysis

**Fig 2:**
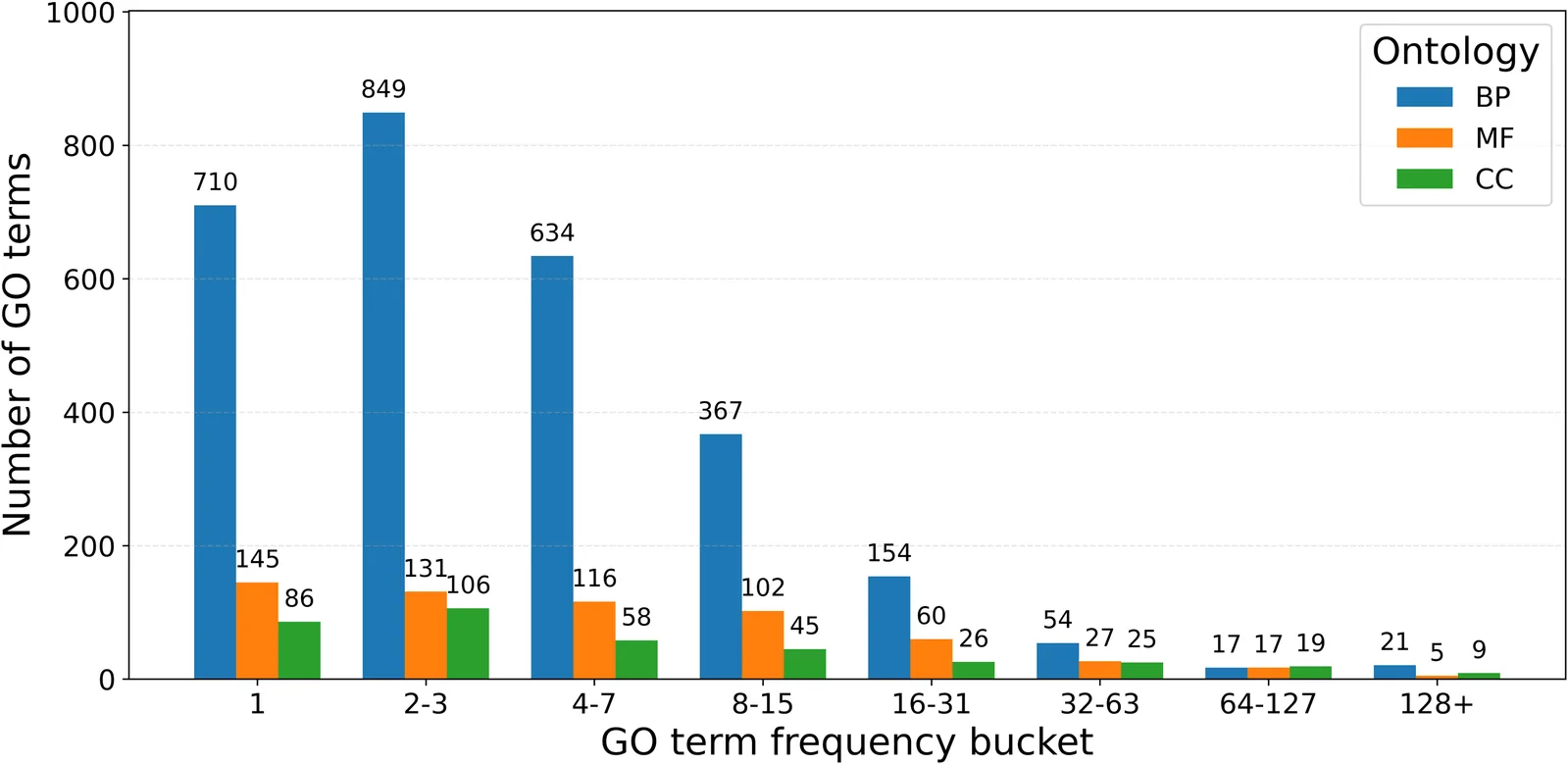
Binned distributions of GO term annotation frequencies in the BP, CC, and MF ontologies of the GPCR dataset, grouped by the number of annotated proteins and highlighting pronounced long-tail behavior and label imbalance.

For the reviewed, labeled GPCR subset partitioned for supervised training, validation, and testing, we summarized GO annotations in terms of term coverage, sequence-length distribution, and average label density (Table 1). BP annotations are missing for a nonnegligible fraction of proteins (13.7%), whereas CC and MF annotations cover nearly the entire dataset. To quantify term specificity, we computed information content (IC) from the direct GO annotations in the final label set, without additional ancestor propagation. Using the natural logarithm, we defined $N_{prot}=3465$ as the total number of proteins in the reviewed, labeled dataset and $n_{prot}(t)$ as the number of distinct proteins annotated with term *t*. The IC of term *t* was calculated as $IC(t)=-\ln\left(\frac{n_{prot}(t)}{N_{prot}}\right)$. We summarized these values at two levels. For the *t*erm-level distribu*t*ion, each unique GO term contributed once. For the annotation-level distribution, each distinct protein–term pair contributed the IC value of its corresponding term, so a term contributed repeatedly according to the number of proteins annotated with it. Because the distributions are skewed, we report the median and interquartile range and define ΔIC as the median of the unique-term distribution minus the median of the annotation-level distribution. Higher IC values correspond to more specific terms, whereas lower IC values indicate more general terms. In this dataset, BP exhibits higher annotation-level IC and lower annotation density than CC, consistent with finer-grained and more long-tailed functional labels. By contrast, CC shows lower IC but higher annotation density, reflecting broader terms with heavier reuse. Between the BP and CC patterns, MF shows intermediate behavior on both axes (Table 1). GO term frequencies also display a pronounced long-tail distribution, as shown in Fig 2. Together, these characteristics indicate a sparse label space and severe class imbalance, placing strong demands on robustness and generalization [27,28].

**Table 1:** Summary statistics for the reviewed, labeled GPCR subset partitioned into training, validation, and test sets, including split sizes, sequence length, branch-specific term and annotation counts, label density, and IC statistics.

Metric	Whole dataset		
Number of proteins	3465		
Training / validation / test split	2772 / 346 / 347 (80.0% / 10.0% / 10.0%)		
Mean sequence length (amino acids)	454		
	BP	CC	MF
Number of unique GO terms	2806	374	603
Total GO annotations	22089	9721	8896
Annotations per term	7.88	25.99	14.75
Mean GO labels per annotated protein	7.38	2.81	2.66
Median annotation-level IC	5.21	3.15	4.09
Interquartile range of annotation-level IC	[3.55, 6.20]	[0.20, 4.26]	[2.93, 5.38]
ΔIC	1.85	3.91	2.67

### Evaluation metrics

To evaluate the performance of different methods on the held-out GPCR test split, we used three evaluation metrics: (i) maximum protein-centric F-score (Fmax), (ii) minimum semantic distance (Smin), and (iii) area under the precision–recall curve (AUPR) [29]. Protein-centric Fmax is defined as the maximum F1 score achieved over prediction thresholds t∈[0,1] with a step size of 0.01. Smin measures the semantic distance between predicted and true annotations while accounting for functional information content. For Smin calculation, the branch-specific IC values computed from the complete reviewed, labeled dataset were fixed and used for both validation and test evaluation. AUPR was calculated in a global micro-averaged manner by flattening the protein–GO label and prediction matrices and constructing a single precision–recall curve over all protein–GO pairs within each GO branch. The validation split was used for model selection, and all final metrics are reported on the independent held-out test split.

To assess how well general protein function predictors transfer to the GPCR family, we first applied the released general-model versions of TALE+ [30] and DeepGO-SE [31] directly to the held-out test split, without GPCR-specific fine-tuning (Table 2). Both models showed low overall performance on GPCRs, and their performance varied markedly across GO branches. For example, in BP, DeepGO-SE (general) achieved an Fmax of 0.041 (AUPR 0.013), indicating limited discriminative ability, whereas TALE+ (general) reached a higher Fmax on CC (0.390) but still degraded substantially on BP (Fmax 0.137). These results indicate that models trained on general protein collections do not transfer readily to GPCRs, especially in BP, where the label space is larger and the long-tail distribution is more pronounced, underscoring the need for GPCR-specific modeling.

**Table 2:** Performance of the released general-model weights of TALE+ and DeepGO-SE on the held-out GPCR test split, reported without GPCR-specific fine-tuning.

	BP			CC			MF		
Model	Fmax	Smin	AUPR	Fmax	Smin	AUPR	Fmax	Smin	AUPR
TALE+ (general)	0.137	99.927	0.049	0.390	17.881	0.194	0.322	16.968	0.154
DeepGO-SE (general)	0.041	220.188	0.013	0.312	29.170	0.108	0.173	23.092	0.056

### Comparative methods

To evaluate GPCR-GO rigorously, we compared it with nine representative baselines: Naive [32], BLAST [33], DeepGOCNN [14], DeepFRI [34], DeepGO-SE [31], TALE+ [30], HEAL [35], GALA [36], and TAWFN [37]. Together, these methods span frequency-based, homology-based, sequence-based, structure-aware, ontology-aware, and graph-based paradigms. Naive relies on label frequency, BLAST transfers annotations by sequence similarity, DeepGOCNN and TALE+ are sequence-centric, DeepFRI and TAWFN integrate structure, DeepGO-SE incorporates ontology-aware constraints, and HEAL and GALA use graph Transformer architectures. Brief descriptions of the baselines are provided below.

The input representations, auxiliary biological information, and training settings used for GPCR-GO and the comparative methods are summarized in Table F in S1 Appendix.

The Naive method predicts GO terms according to their frequencies in the training set, ignoring sequence and structural information. It serves as a simple frequency-based reference under the same evaluation protocol.

The BLAST method predicts functions for each query protein in the held-out split by searching the training set with BLASTP under a specified E-value threshold and transferring annotations from the most similar matched sequence. As a homology-based strategy, it provides a classical sequence-similarity baseline.

DeepGOCNN uses convolutional neural networks to learn local motif and contextual patterns directly from protein sequences and then maps the resulting sequence representations to GO term predictions. By emphasizing discriminative subsequence features, it provides an efficient sequence-based baseline for large-scale protein function annotation.

DeepFRI combines graph convolutional networks with sequence and three-dimensional structural information. It uses a long short-term memory network to extract residue-level sequence embeddings and propagates them through multiple graph convolution layers on protein contact graphs. With global pooling and fully connected layers, DeepFRI predicts GO terms and Enzyme Commission numbers, and it further employs gradient-weighted class activation mapping to interpret predictions and identify key residues.

DeepGO-SE performs prediction in geometric semantic spaces defined over GO ontologies and introduces ontology-aware logical constraints for protein function inference. By jointly embedding proteins and GO classes in these semantic spaces, it improves ontology consistency during annotation.

TALE+ extends the TALE framework by strengthening joint modeling of protein sequences and label semantics with Transformer-based encoders. Through label-aware sequence representation learning and enhanced sequence–label interaction, it improves annotation for proteins with limited homology and serves as a strong sequence-based baseline.

HEAL addresses the difficulty of modeling long-range spatial dependencies in conventional graph neural networks by using a hierarchical graph Transformer. It introduces learnable semantic super-nodes to capture global topological information and interacts with residue nodes through attention to aggregate functional substructures. Graph contrastive learning is used as a regularizer to improve robustness and discrimination.

GALA combines a graph Transformer, multi-head attention, and learnable meta-nodes to model global semantics and integrate local structure with global topology effectively. An attention-pooling module aggregates meta-node representations to produce more discriminative protein embeddings. GALA further introduces adversarial learning and label-embedding alignment to enhance transfer to low-homology proteins and improve semantic consistency.

TAWFN proposes a dual-model adaptive weight-fusion network that integrates convolutional neural network and graph convolutional network components to capture structural and sequence information jointly. Through an adaptive graph convolution module and long-range dependency modeling with a long short-term memory network, the model aggregates local geometric information in the structural branch while strengthening global semantics through multi-head attention. Adaptive fusion then dynamically reweights structural and sequence features to improve coordination and robustness in representation learning and decision making.

### GPCR-GO improves protein function prediction

**Fig 3:**
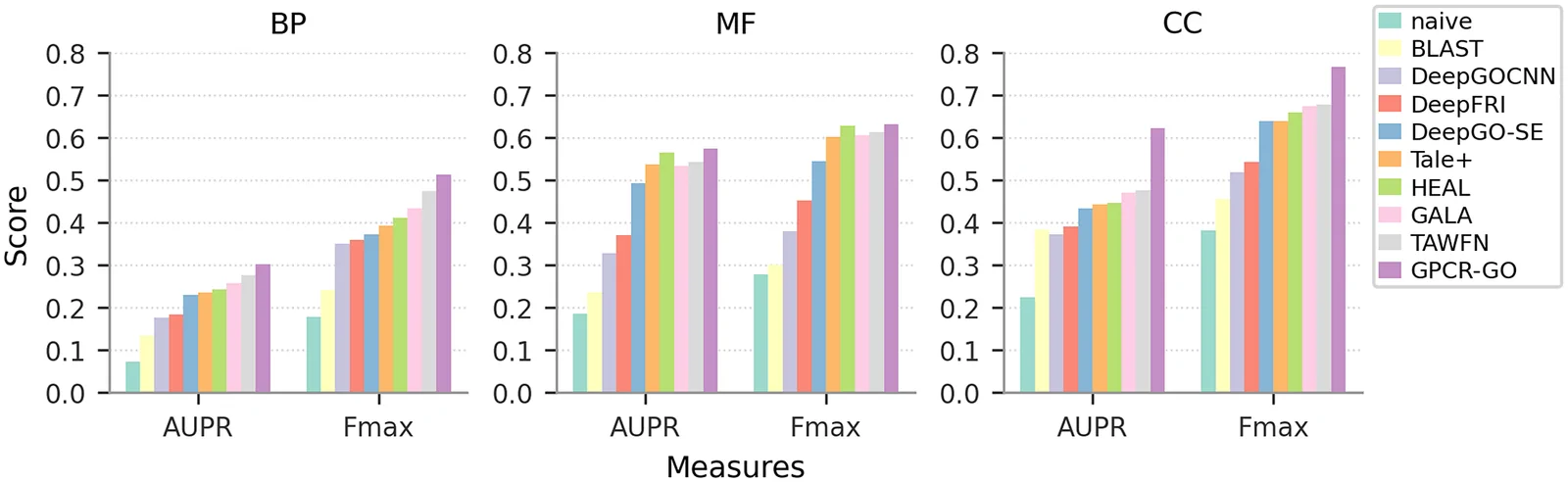
Comparison of AUPR and Fmax across methods on the held-out GPCR test split.

We evaluated GPCR-GO on the held-out GPCR test split against nine baselines: Naive, BLAST, DeepGOCNN, DeepFRI, DeepGO-SE, TALE + , HEAL, GALA, and TAWFN. All baselines in Table 3, including GPCR-retrained versions of DeepGO-SE and TALE + , were retrained on the same GPCR split used for GPCR-GO. Performance was evaluated independently for BP, CC, and MF. GPCR-GO achieves Fmax values of 0.514, 0.767, and 0.631 on BP, CC, and MF, with corresponding Smin values of 16.374, 3.622, and 4.572 and AUPR values of 0.302, 0.623, and 0.575. It outperforms all competing methods across the three GO branches on this held-out test split, indicating greater stability under the fixed evaluation protocol. Relative to strong deep-learning baselines such as HEAL, GALA, and TAWFN, GPCR-GO shows clearer gains in both Fmax and AUPR, indicating a better precision–recall trade-off under severe class imbalance. GPCR-GO also yields lower Smin values, suggesting that its predictions are semantically closer to the true annotations and better aligned with the GO hierarchy. These comparative trends are also visualized in Fig 3. Additional multi-seed results across BP, CC, and MF are reported in Table A in S1 Appendix.

**Table 3:** Fmax, Smin, and AUPR of different methods on the held-out GPCR test split.

	BP			CC			MF		
Model	Fmax	Smin	AUPR	Fmax	Smin	AUPR	Fmax	Smin	AUPR
Naive	0.178	34.786	0.072	0.381	8.917	0.225	0.278	11.591	0.185
BLAST	0.241	29.339	0.134	0.455	7.844	0.384	0.301	10.241	0.236
DeepGOCNN	0.351	20.574	0.176	0.518	5.772	0.373	0.380	8.176	0.328
DeepFRI	0.359	19.362	0.184	0.542	5.327	0.391	0.452	7.161	0.371
DeepGO-SE	0.373	17.854	0.230	0.639	4.663	0.434	0.545	6.563	0.492
TALE+	0.392	17.348	0.235	0.640	4.588	0.442	0.603	5.968	0.537
HEAL	0.411	16.618	0.243	0.659	4.420	0.446	0.629	5.426	0.565
GALA	0.433	14.384	0.257	0.674	4.162	0.470	0.605	5.839	0.534
TAWFN	0.475	**13.898**	0.277	0.679	4.253	0.476	0.613	5.532	0.542
GPCR-GO-Sup	0.508	18.583	0.273	0.749	4.815	0.603	0.622	5.160	0.553
GPCR-GO	**0.514**	16.374	**0.302**	**0.767**	**3.622**	**0.623**	**0.631**	**4.572**	**0.575**

### Hyperparameter settings

Throughout the manuscript, we use $P_{e}$ for the negative sampling ratio, $P_{n}$ for the positive-sample weight, $P_{h}$ for the hard-negative proportion, and λ for the shared regularization weight in the graph decomposition objective.

### Negative sampling ratio and positive sample weight

**Fig 4:**
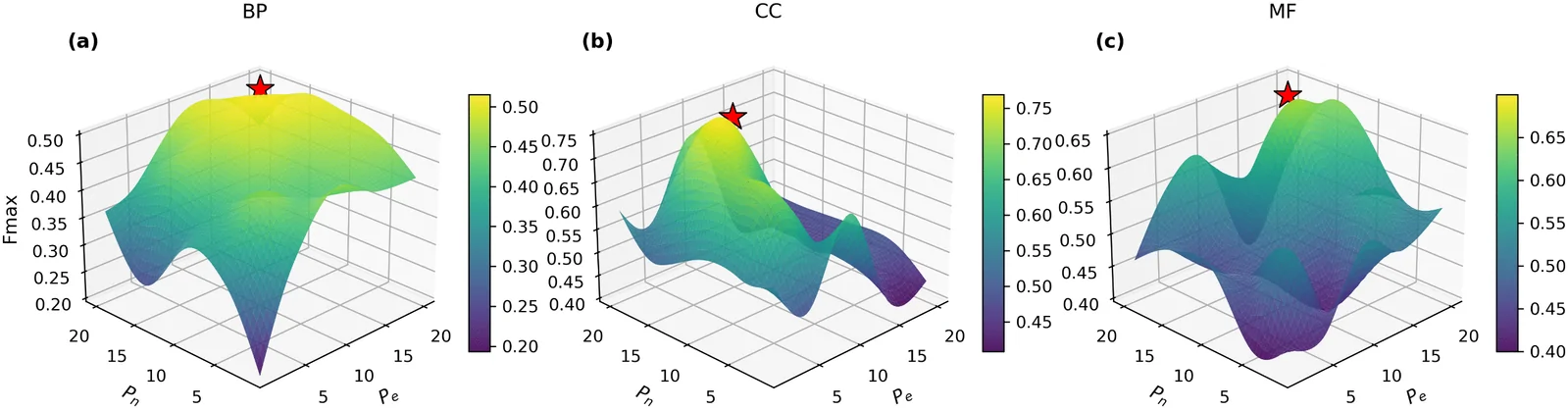
Effects of the negative sampling ratio ($P_{e}$) and positive-sample weight ($P_{n}$) on Fmax in the heterogeneous-graph semi-supervised setting. The figure shows test-set Fmax surfaces for BP, CC, and MF on a two-dimensional hyperparameter grid (x-axis $P_{e}$, y-axis $P_{n}$), with height and color encoding Fmax. Red stars mark the optimal parameter combinations. Grid search shows that the best settings are ($P_{n}=15$, $P_{e}=15$) for BP (Fmax 0.514), ($P_{n}=15$, $P_{e}=10$) for CC (Fmax 0.767), and ($P_{n}=15$, $P_{e}=15$) for MF (Fmax 0.631).

The negative sampling ratio $P_{e}$ and the positive-sample weight $P_{n}$ are two key hyperparameters in the heterogeneous-graph semi-supervised setting. Specifically, $P_{e}$ controls the ratio of sampled negative examples for reviewed proteins during graph training, whereas $P_{n}$ controls the weight assigned to positive samples in the weighted binary cross-entropy (BCE) loss. Their joint tuning is important because they play complementary roles. Negative sampling mitigates the imbalance between positive and negative instances by selecting only a subset of negatives for training, thereby reducing overfitting to abundant negatives and promoting broader learning while keeping unreviewed proteins label-free. However, negative sampling alone may still be insufficient when negatives overwhelmingly outnumber positives. In that regime, the positive-sample weight becomes critical because it increases the influence of sparse but biologically important positive annotations and improves learning under class imbalance. In this analysis, the hard-negative proportion $P_{h}$ was fixed, and only $P_{e}$ and $P_{n}$ were varied in a sensitivity analysis on the held-out test split. As shown in Fig 4, the best combinations were $P_{n}=15$ and $P_{e}=15$ for BP (Fmax 0.514), $P_{n}=15$ and $P_{e}=10$ for CC (Fmax 0.767), and $P_{n}=15$ and $P_{e}=15$ for MF (Fmax 0.631). To assess whether the conclusions depended on a particular hard-negative setting, we further compared random-only sampling ($P_{h}=0$), mixed hard/random sampling, and hard-only sampling ($P_{h}=1.0$). These analyses showed that performance was not driven by hard-only sampling or by a single hard-negative configuration, supporting the robustness of the main conclusions to the hard-negative proportion. Detailed results are reported in Table G in S1 Appendix.

### Number of factors and regularization weight in the graph decomposition network

To improve representation separability under heterogeneous relation stacking, we introduce a Graph Decomposition Network (GDN) on top of the encoder outputs, splitting each node representation along the feature dimension into *M* factor subspaces to obtain relatively independent yet complementary semantic representations. In addition to the main-task BCE loss, we introduce the two regularizers defined in the Loss Function Optimization section—inter-factor node repulsion and inter-factor space separation—to suppress redundancy and collapse at different levels, and combine them through weighted summation into the final training objective. Because both regularizers promote complementarity and independence among factors, we tie their weights to a single hyperparameter λ, that is, $\lambda_{1}=\lambda_{2}=\lambda$, to reduce the number of free parameters and improve the reproducibility and interpretability of tuning.

When λ=0, both regularization terms are removed from the objective function, and GDN factorization no longer constrains parameter updates. In this degenerate case, as long as an equivalent mapping is preserved in the absence of additional perturbations or constraints, the learning objective and the resulting predictions typically remain unchanged. Under such circumstances, *M* no longer affects optimization directly and primarily influences computation and memory cost. Therefore, only when λ>0 does *M* interact with the structural inductive bias and regularization to influence representation learning and predictive performance.

**Fig 5:**
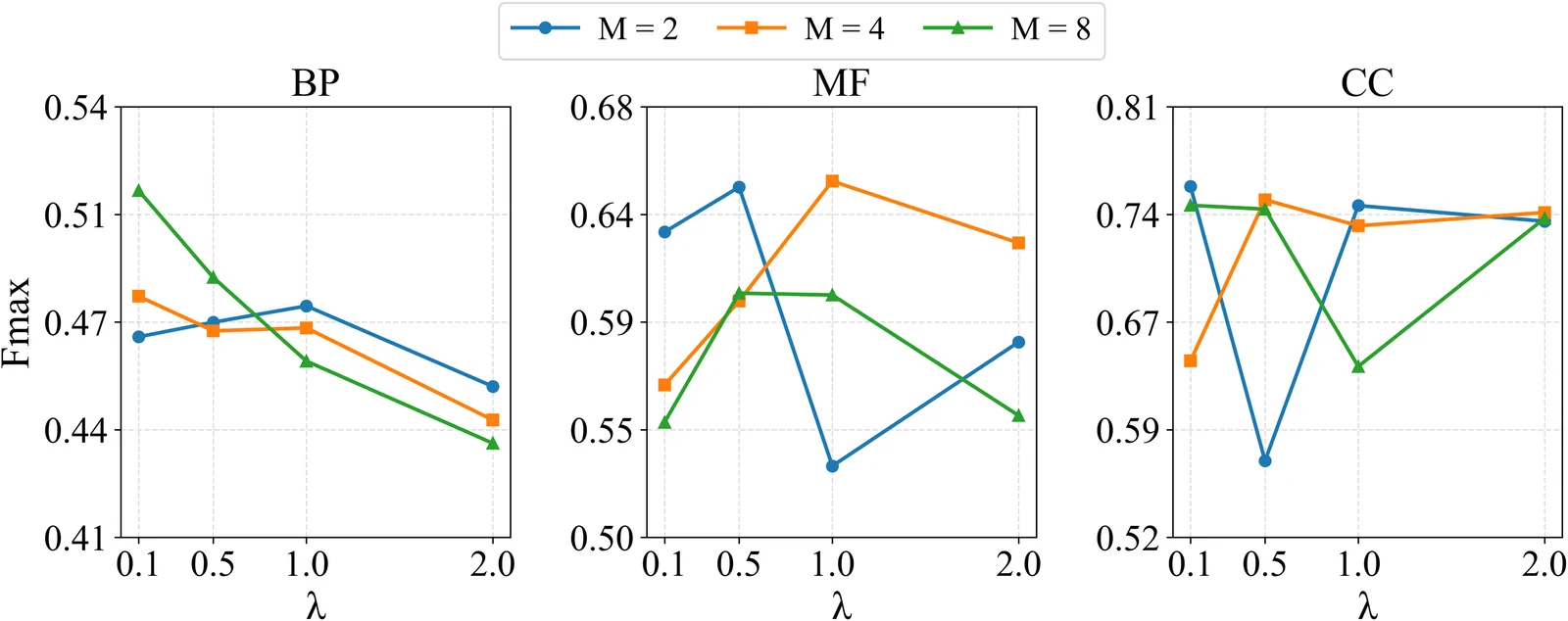
Effects of the regularization weight λ and the factor number *M* on Fmax. The x-axis represents λ, the y-axis represents Fmax, and different colored curves correspond to different values of *M*. Results are shown for BP, CC, and MF.

Keeping all other parameters fixed, we evaluated *M* and λ in a sensitivity analysis on the held-out test split. Fig 5 shows how Fmax varies with *M* and λ for BP, CC, and MF. The three branches display different sensitivities. BP tends to decrease as λ increases, indicating greater sensitivity to regularization, whereas larger *M* values help decompose complex semantics more finely. CC is relatively robust overall, but smaller *M* values are advantageous, suggesting that overly fine factorization may weaken aggregated representations. MF benefits more stably from moderate λ values and often prefers an intermediate *M*. The final GPCR-GO configuration used the following branch-specific settings: BP used *M* = 8 and λ=0.1, CC used *M* = 2 and λ=0.1, and MF used *M* = 4 and λ=1.0. The complete branch-specific hyperparameter configuration evaluated on the held-out test split is summarized in Table L in S1 Appendix.

### Ablation study

We performed single-component ablations of semi-supervised learning (SSL), structural-similarity edges (SSE), graph decomposition, and hard negative mining (HNM). Removing any component reduced performance across BP, CC, and MF (Table 4). The largest drop occurred without HNM, especially for BP and CC, suggesting that difficult negative training pairs help improve discrimination under label imbalance. Removing SSL or SSE also consistently reduced Fmax and AUPR, and removing GDN caused the largest MF decline, indicating that all four modules contribute complementary gains.

**Table 4:** Ablation of SSL, SSE, GDN, and HNM on the GPCR test split.

	BP			CC			MF		
Model	Fmax	Smin	AUPR	Fmax	Smin	AUPR	Fmax	Smin	AUPR
GPCR-GO w/o HNM	0.388	27.854	0.173	0.592	7.059	0.356	0.513	7.116	0.470
GPCR-GO w/o SSE	0.486	21.082	0.257	0.738	5.150	0.598	0.626	6.098	0.549
GPCR-GO w/o GDN	0.496	20.670	0.265	0.729	5.241	0.600	0.588	7.568	0.491
GPCR-GO w/o SSL	0.508	18.583	0.273	0.749	4.815	0.603	0.622	5.160	0.553
GPCR-GO	**0.514**	**16.374**	**0.302**	**0.767**	**3.622**	**0.623**	**0.631**	**4.572**	**0.575**

Additional analyses showed that SSE-connected protein pairs had greater GO-annotation overlap than PPI and endpoint-matched random controls (Table B in S1 Appendix). Protein-level effects were heterogeneous and were not significantly associated with PPI degree (Table D in S1 Appendix).

### Case study

To further assess the predictive capacity of GPCR-GO at the level of individual proteins, three representative proteins, O77713, P47898, and P46092, were selected from the held-out test results for case-study analysis. For each protein, experimentally annotated and predicted GO terms were compared across the three GO sub-ontologies: biological process (BP), cellular component (CC), and molecular function (MF). Predicted terms were taken from the ranked model outputs on the held-out test split, and true annotations appearing in the reported prediction lists are highlighted in bold in Table 5. Unlike global performance metrics, this analysis focuses on whether the model can recover key functional terms for specific proteins, whether it can provide biologically plausible complementary annotations, and where its ranking boundaries emerge. As shown in Table 5, the three proteins collectively contain 34 true GO terms, 33 of which were recovered in the reported predictions.

**Table 5:** Case-study comparison between true GO annotations and predicted GO terms for three representative proteins from the held-out test split. True annotations recovered in the reported prediction lists are highlighted in bold.

Protein entry	GO aspect	True GO annotations	Predicted GO terms
O77713	BP	GO:0030168; GO:0019229; GO:0006940	**GO:0030168**; **GO:0006940**; GO:0071881; **GO:0019229**
	CC	GO:0005886; GO:0009986	**GO:0005886**; **GO:0009986**
	MF	GO:0004938; GO:0051379	**GO:0051379**; **GO:0004938**
P47898	BP	GO:0007186; GO:0007189; GO:0007268; GO:0007187; GO:0032355; GO:0007198; GO:0021766	**GO:0007186**; **GO:0007187**; **GO:0007268**; **GO:0007189**; **GO:0007198**; GO:0019222; GO:0007165; **GO:0021766**; **GO:0032355**; GO:0007191
	CC	GO:0005886; GO:0030425; GO:0043204; GO:0099634	**GO:0005886**; **GO:0030425**; GO:0045202; **GO:0043204**; GO:0045211; GO:0030424; GO:0042734; GO:0098685; GO:0098793; GO:0043025
	MF	GO:0004993; GO:0030594; GO:0001586; GO:0099589	**GO:0004993**; **GO:0030594**; **GO:0099589**; GO:0051378; **GO:0001586**; GO:0004969; GO:0004930; GO:0016907; GO:0090722; GO:0001594
P46092	BP	GO:0007186; GO:0019722; GO:0060326; GO:0006955; GO:0007204	**GO:0007186**; **GO:0007204**; **GO:0006955**; **GO:0019722**; GO:0006935; **GO:0060326**; GO:0006954
	CC	GO:0005886; GO:0009897; GO:0005783; GO:0009986	**GO:0005886**; **GO:0009986**; **GO:0009897**; GO:0005769; GO:0055037; **GO:0005783**
	MF	GO:0004930; GO:0019957; GO:0016493	**GO:0004930**; **GO:0016493**; **GO:0019957**; GO:0071791

For O77713, the model showed a high degree of consistency across all three sub-ontologies. In BP, GO:0030168, GO:0019229, and GO:0006940 were accurately predicted; in CC, GO:0005886 and GO:0009986 were correctly identified; and in MF, GO:0004938 and GO:0051379 were fully identified. In addition to the true annotations, the model predicted GO:0071881, which denotes an adrenergic receptor-mediated inhibitory adenylate cyclase signaling pathway. This term is highly consistent with the known receptor activity and ligand-binding function of the protein, suggesting that GPCR-GO can identify not only explicit functional labels but also downstream signaling characteristics.

For P47898, the model recovered all annotated BP and MF terms within the top-ranked predictions. At the BP level, the recovered terms included GPCR signaling-related processes (GO:0007186, GO:0007187, GO:0007189, and GO:0007198), chemical synaptic transmission (GO:0007268), response to estradiol (GO:0032355), and hippocampus development (GO:0021766). In the CC branch, GPCR-GO recovered plasma membrane (GO:0005886), dendrite (GO:0030425), and perikaryon (GO:0043204), but did not rank postsynaptic specialization membrane (GO:0099634) within the top 10. Instead, the remaining high-ranked CC predictions were mainly associated with broader neuronal and synaptic localization. At the MF level, GPCR serotonin receptor activity (GO:0004993), neurotransmitter receptor activity (GO:0030594), serotonin receptor activity (GO:0099589), and Gi/o-coupled serotonin receptor activity (GO:0001586) were all ranked in the top 10 predictions. We therefore retained P47898 as a partially successful case: GPCR-GO accurately captured the signaling processes, major neuronal localization, and receptor function of this protein, but failed to rank the more specific postsynaptic specialization membrane term within the top 10.

For P46092, the prediction results also exhibited strong biological consistency. All 12 true GO annotations of this protein were successfully recovered, including BP terms GO:0007186, GO:0019722, GO:0060326, GO:0006955, and GO:0007204; CC terms GO:0005886, GO:0009897, GO:0005783, and GO:0009986; and MF terms GO:0004930, GO:0019957, and GO:0016493. Importantly, the additionally predicted terms GO:0006935 and GO:0006954 correspond to chemotaxis and inflammatory response, respectively, both of which are closely related to the immune-cell migration functions mediated by C-C chemokine receptors. Moreover, GO:0005769 and GO:0055037 are associated with early endosomes and recycling endosomes, suggesting that the model further captured subcellular localization features related to receptor internalization and recycling. Although GO:0071791 denotes a more specific chemokine ligand-binding term and requires further experimental validation, this prediction remains functionally consistent with the chemokine receptor identity of the protein at the family level.

Overall, these three case studies demonstrate that GPCR-GO can recover most core GO annotations and generate complementary terms that are closely related to known protein functions. In particular, the predictions showed strong consistency in receptor activity, signal transduction, immune-related processes, and membrane localization. The P47898 case further illustrates that remaining errors may arise from ranking highly specific subcellular localization terms below more general neuronal or synaptic compartments. This finding is consistent with the case-study strategy adopted in DeepSS2GO, which emphasizes whether key functional terms are correctly identified [38]. Thus, the case-study analysis supports the practical utility of GPCR-GO for recovering core annotations at the single-protein level, while also showing that highly specific GO terms may remain difficult to rank correctly.

## Discussion

This study addresses GPCR function prediction in a small-sample regime and introduces GPCR-GO, a relation-aware heterogeneous graph attention model that integrates structural similarity, PPI networks, and GO ontology information. The main difficulty arises from the scarcity of experimentally validated annotations together with a strongly long-tailed label space, which makes accurate decision-boundary learning difficult, particularly for low-frequency labels. The limited transferability of general protein function predictors further emphasizes the distinct challenges of the GPCR family.

GPCR-GO addresses these constraints through four coordinated components. Structural-similarity edges provide an additional source of protein-protein relatedness that is distinct from PPI neighborhoods and can enrich message-passing paths for a subset of proteins. Semi-supervised learning incorporates large numbers of unlabeled proteins into the heterogeneous graph, allowing annotation signals to diffuse through multi-relation neighborhoods. Hard negative mining selects high-scoring candidate protein–GO pairs as negative training examples, sharpening decision boundaries and reducing the dominance of easy negatives under imbalanced supervision. The graph decomposition network disentangles node representations into low-redundancy factor subspaces, mitigating semantic entanglement introduced by multi-source fusion.

These modules act as a coordinated system rather than a simple stack: structural-similarity edges enrich propagation paths, semi-supervised learning exploits those paths to diffuse supervision, hard negative mining sharpens discrimination in richer neighborhoods, and graph decomposition provides clearer representations for all three processes. Ablation analysis shows that removing any one of them reduces performance, supporting both the necessity of each component and the value of their synergy.

Across all three GO branches, GPCR-GO substantially outperforms existing methods on the held-out test split, indicating improved robustness under small-sample and imbalanced conditions. Accordingly, we interpret these gains primarily as improved overall robustness under sparse and imbalanced supervision, rather than as uniform improvement for every low-frequency GO term. A frequency-stratified analysis further showed substantially lower recovery for rare and unseen GO terms (Table C in S1 Appendix), reinforcing that the overall gains should not be interpreted as uniform performance across label-frequency strata. Several limitations nevertheless remain. First, GO function prediction is commonly formulated in a positive–unlabeled setting, because experimentally verified absence of a protein–GO association is usually unavailable. As in many protein function prediction studies, GPCR-GO uses non-positive reviewed protein–GO pairs as the candidate pool for sampled negative examples. This issue is particularly relevant for hard negative mining, because high-scoring candidate pairs near the decision boundary may include biologically present functions that have not yet been curated, thereby introducing false-negative bias. The sensitivity analyses above reduce, but do not eliminate, this concern. Future work should explore positive–unlabeled objectives and ontology-aware negative filtering to further reduce this source of uncertainty. In practical use, GPCR-GO scores are intended to rank candidate protein–GO associations for further inspection rather than to replace established GO evidence. As in other multi-label protein function prediction tasks, threshold selection determines the balance between recall and precision: lower thresholds recover more candidate terms but may increase false positives, whereas higher thresholds provide more conservative predictions but may miss some true annotations. Threshold-dependent precision, recall, F1, and micro- and macro-averaged results obtained with validation-selected thresholds are provided in Table I in S1 Appendix. The quality of structure-derived similarity edges may be affected by the confidence and completeness of available protein structures, and the Top-*K* edge-selection strategy may require further sensitivity analysis across datasets. In addition, because experimentally annotated reviewed GPCRs remain limited and were comprehensively collected from UniProt in this study, a fully independent labeled GPCR benchmark is not readily available. Future work will therefore evaluate temporal annotation updates, sequence-cluster-based splits, and subfamily-level generalization to further assess robustness beyond random internal partitions.

## Materials and methods

### Dataset construction

This study focuses on GPCR function prediction. We retrieved GPCR-related entries from UniProt, comprising 3,465 reviewed sequences and 849,781 unreviewed sequences [39]. Because the reviewed set is limited, only reviewed proteins were treated as label-bearing instances with GO supervision, whereas unreviewed proteins were included solely as unlabeled nodes to support semi-supervised learning and improve generalization [40]. Under this design, GO supervision is defined exclusively on the reviewed subset: observed positive annotations provide protein–GO edges in the heterogeneous graph, whereas sampled negative protein–GO pairs are generated only for decoder-level training supervision. Unreviewed proteins participate only in graph message passing and are never assigned GO labels. This choice is methodologically important because many function labels are available in a positive–unlabeled regime; treating unreviewed proteins or their unlabeled GO terms as negatives would risk introducing false-negative bias [41]. Among the unreviewed entries, we further filtered proteins by UniProt annotation score (1–5) [42], retaining 3,364 entries with score 5, 4,771 with score 4, and 51,914 with score 3, yielding 60,049 candidate unlabeled GPCR entries for subsequent semi-supervised graph construction.

To minimize information leakage among training, validation, and testing data, we randomly split the reviewed, labeled subset into training, validation, and test sets at an 8:1:1 ratio, yielding 2,772, 346, and 347 proteins, respectively. The validation set was used for model selection and early stopping, whereas all final quantitative results reported here were obtained on the independent held-out test set. Although this protocol provides an internal train/validation/test evaluation, the reported results should still not be interpreted as estimates from an external benchmark. To reduce redundancy in the supplemental unlabeled pool, we clustered the filtered unreviewed sequences with CD-HIT at a 90% similarity threshold and retained one representative sequence per cluster, thereby limiting near-duplicate unlabeled nodes that could otherwise overemphasize duplicate signals during graph construction and message passing [43,44]. After deduplication, 24,212 nonredundant unlabeled GPCR sequences remained for graph-based semi-supervised modeling. The final graph used by GPCR-GO therefore comprises two layers of data: a reviewed, labeled subset of 3,465 proteins for supervision and evaluation, and a supplemental unlabeled subset of 24,212 proteins for representation learning and information propagation.

### GO annotation and label construction

Protein–function pairs were constructed according to the Gene Ontology (GO) annotation system under the reviewed-protein supervision setting. GO annotations were extracted from UniProtKB GPCR reviewed entries downloaded on 2025-08-21. The GO ontology file used for term checking and ontology-edge construction was go.obo release 2023-04-01. Specifically, positive samples were defined as protein–GO term pairs derived from the GO annotations of reviewed proteins, and the observed positive annotations were used to construct protein–GO edges in the heterogeneous graph. During training, negative protein–GO pairs were sampled for reviewed proteins by selecting GO terms that were not assigned as positive annotations to the same protein. These sampled pairs were used only as negative targets for decoder optimization; they were not inserted into the heterogeneous graph. Moreover, they should not be interpreted as curated biological evidence that a function is absent.

GO provides a unified vocabulary spanning biological process (BP), cellular component (CC), and molecular function (MF) [45]. As in previous work, we constructed separate datasets for the three branches and evaluated each branch independently, enabling branch-specific assessment of model behavior. GO hierarchical relations, including *is_a* and *part_of* relations, were used to construct GO–GO ontology edges in the heterogeneous graph.

### Protein-Protein Interaction Network (PPI)

In this study, PPI edges were derived from the BioGRID interaction database [46]. Specifically, we used BIOGRID-ALL-4.4.243.tab3.txt and retained physical interactions for which both endpoints could be mapped to GPCR proteins in the constructed graph. Each retained interaction was encoded as a protein-protein edge with relation type 1 and edge weight 1.0. In the final branch-specific graphs, this procedure yielded 3,055 PPI edges for each GO branch. These PPI edges provide additional biological context beyond sequence and structure alone by enabling message passing between interacting proteins [47].

### Problem statement

Given a set of GPCR proteins $𝒫={𝒫}_{r}\cup {𝒫}_{u}$, where ${𝒫}_{r}$ denotes reviewed proteins with GO annotations and ${𝒫}_{u}$ denotes unreviewed proteins without supervision, we formulate GPCR function prediction as a branch-specific multi-label protein–GO association prediction problem. For each GO branch c∈{BP,CC,MF}, let ${𝒢}^{c}$ be the set of candidate GO terms and let ${𝐲}_{i}^{c}\in \{0,1{\}}^{\left|{𝒢}^{c}\right|}$ denote the annotation vector of reviewed protein $p_{i}\in {𝒫}_{r}$. Here, $y_{ij}^{c}=1$ indicates that protein $p_{i}$ is annotated with GO term $g_{j}^{c}$ in the final GO label set, whereas $y_{ij}^{c}=0$ denotes only that this association is not included in the curated annotations, not that the function is biologically absent. During training, negative protein–GO pairs are sampled only from these non-positive reviewed-protein associations, as described below. Thus, GO supervision is restricted to ${𝒫}_{r}$.

The objective is to learn a branch-specific scoring function $f_{c}(p_{i},g_{j}^{c};ℋ)\to [0,1]$ on a heterogeneous graph ℋ containing protein nodes, GO term nodes, protein-protein interaction edges, structural-similarity edges, GO hierarchical edges, and training protein–GO annotation edges constructed only from reviewed proteins. For evaluation, the model scores candidate pairs $\left(p_{i},g_{j}^{c}\right)$ for reviewed proteins in the held-out split, and outputs a confidence score ${\hat{y}}_{ij}^{c}$ indicating the probability that protein $p_{i}$ should be associated with GO term $g_{j}^{c}$. Final prediction is therefore a branch-specific multi-label ranking or classification task performed separately for BP, CC, and MF.

### Model architecture and implementation

The following sections describe the sequence encoder, structural-similarity graph construction, heterogeneous graph attention module, graph decomposition component, negative sampling strategy, and training objective used in GPCR-GO. A unified list of symbols, abbreviations, notation, and model dimensions is provided in Table J in S1 Appendix.

## Protein representation

### Protein sequence feature extraction

Large protein language models have recently transformed sequence representation learning. ESM2 is widely used because it delivers strong contextual embeddings across diverse protein analysis tasks. Built on the Transformer architecture, ESM2 captures both local residue context and long-range sequence dependencies through self-attention. The model is pre-trained in a self-supervised manner on millions of protein sequences and therefore encodes rich evolutionary and structure-related information.

For an input protein sequence $s=(s_{1},...,s_{N})$, ESM2 first tokenizes each amino-acid residue and then processes the sequence through multiple Transformer layers to obtain a contextual embedding for each residue. For residue *k*, the feature vector is defined as:


$$\begin{array}{c} {𝐡}_{k}=Transformer\;{\left(Embed(s)\right)}_{k}. \end{array}$$ (1)

Here, Embed(s) denotes the sequence of initial residue embeddings, and ${𝐡}_{k}$ is the final contextual representation of residue *k*. Concatenating all residue features yields a protein sequence feature matrix of dimension N×D:


$$\begin{array}{c} 𝐗=[{𝐡}_{1},...,{𝐡}_{N}]\in {\mathbb{R}}^{N\times D}. \end{array}$$ (2)

In this study, we use the pre-trained protein language model esm2_t33_650M_UR50D to extract protein sequence features. For each GPCR sequence, the resulting ESM2 feature vector is used in downstream function prediction.

### Structural similarity edges

**Fig 6:**
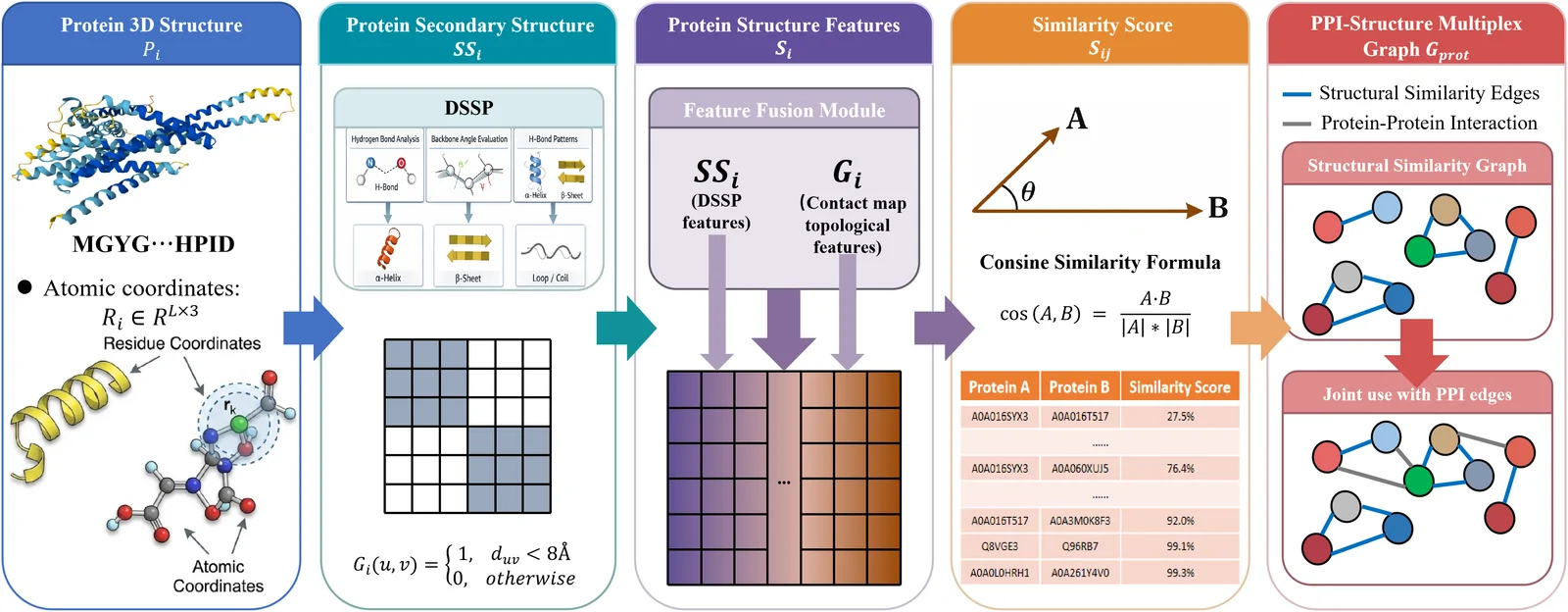
Workflow of SSE construction. Local residue neighborhoods are defined using an 8 Å contact rule, DSSP-derived residue descriptors are extracted from each three-dimensional protein structure, feature-wise mean/std pooling yields a 26-dimensional protein-level vector ${𝐳}_{i}$, pairwise similarity scores $S_{ij}$ are computed, and top-$K_{SSE}$ selection produces the final structural-similarity graph.

To characterize structural similarity between proteins, we construct structural-similarity edges with a dedicated module (Fig 6). Three-dimensional structures were collected preferentially from the Protein Data Bank (PDB). AlphaFold Protein Structure Database models were used only when no experimental PDB structure was available. For AlphaFold-derived structures, we retained only models with mean predicted local distance difference test (pLDDT) score ≥70 (equivalently, normalized pLDDT ≥0.7). Structures below this threshold were excluded from structural-similarity-edge construction to reduce noise from low-confidence structural predictions. Proteins without reliable structures were not removed from the heterogeneous graph; instead, they were retained as protein nodes and could still contribute through sequence features, PPI relations, and, for reviewed proteins, GO annotation supervision. These proteins were simply not assigned structural-similarity edges. For each protein structure, residue-residue contacts were defined with an 8 Å cutoff to characterize local structural neighborhoods. DSSP was then used to extract residue-level structural descriptors. In the preprocessing pipeline used for edge construction, each residue is represented by a 13-dimensional vector comprising sin(ϕ), cos(ϕ), sin(ψ), cos(ψ), relative solvent accessibility, and an 8-state DSSP secondary-structure one-hot encoding. To obtain a fixed-length protein representation for pairwise comparison, we pooled the residue descriptor matrix by concatenating the feature-wise mean and standard deviation across residues, yielding a 26-dimensional protein-level vector ${𝐳}_{i}$.

After obtaining structural vector representations, we compute the structural similarity score $S_{ij}$ (based on cosine similarity) for any protein pair (*i*,*j*):


$$\begin{array}{c} S_{ij}=\frac{{𝐳}_{i}\cdot {𝐳}_{j}}{\|{𝐳}_{i}{\|}_{2}\|{𝐳}_{j}{\|}_{2}}. \end{array}$$ (3)

To control the density of the structural graph and keep it comparable in scale to the PPI graph, we retained the top-$K_{SSE}$ protein pairs with the highest structural similarity scores, with $K_{SSE}=3000$. The PPI graph contained 3,055 edges; therefore, this setting produced an SSE graph with an edge count close to, but not exactly equal to, that of the PPI graph. This approximate density calibration limits graph sparsity while avoiding a substantial imbalance between structural-similarity and interaction information. It also filters for relations with stronger biological relevance, thereby reducing redundancy, noise, and oversmoothing in an overly dense structural graph. With this PPI-comparable density setting, the model can exploit PPI interactions while still benefiting from efficient propagation of structural information.

The resulting structural similarity graph introduces complementary three-dimensional constraints without substantially increasing graph density. These edges provide protein-protein relatedness that is not captured by curated interaction networks and may offer additional propagation paths for proteins whose interaction neighborhoods are limited.

### Heterogeneous graph construction

The node set 𝒱 comprises two node types: proteins and GO terms. Protein nodes include reviewed proteins with GO supervision and unreviewed proteins used only as unlabeled nodes for semi-supervised propagation. Both subsets pass through the same sequence-encoding and structure-based edge-construction pipeline; however, only reviewed proteins are connected to GO terms through annotation edges and contribute supervised labels. For protein nodes (denoted by ${𝒱}_{p}$), high-dimensional sequence features extracted from pre-trained models such as ESM2 serve as initial node attributes ${𝐱}_{i}^{p}$, capturing sequence information and biological semantics. GO term nodes (denoted by ${𝒱}_{g}$) are initialized with one-hot vectors ${𝐱}_{j}^{g}$ representing their unique identities in the ontology. Because protein and GO nodes have different feature dimensions, we project them into a shared latent space using a linear transformation.

The edge set ℰ encodes four biological relation categories: protein–GO annotation edges, BioGRID-derived PPI edges, structural-similarity edges, and GO-GO hierarchy edges. Protein–GO annotation edges are encoded as relation type 0 and are used as the supervised prediction target. PPI edges are encoded as relation type 1, structural-similarity edges as relation type 2, and GO-GO hierarchy edges as relation type 3. GO-GO hierarchy edges were parsed from go.obo using both *is_a* and *part_of* parent-child relations. These two ontology relations were not separated into different edge types; instead, both were encoded as a single GO-GO hierarchy relation. The original GO-GO edge direction is from child GO term to parent GO term. During graph construction, reverse edges and self-loops are added for message passing, so ontology information can propagate bidirectionally while preserving relation-type identities. In the final graphs, the numbers of GO-GO hierarchy edges were 781 for BP, 166 for CC, and 236 for MF. Unreviewed proteins participate through the same protein–protein and structural-similarity relations as reviewed proteins, but they do not carry protein–GO annotation edges. By integrating these relation types, the heterogeneous graph provides a unified representation of complementary biological knowledge for downstream inference.

### Heterogeneous graph attention

Graph attention networks (GAT) [48] are well suited to graph-structured data because they adaptively assign attention weights to neighboring nodes during feature aggregation. Relative to conventional graph convolutional networks, GAT can modulate information flow according to relation-specific context, which improves representation learning on complex graphs. In protein function prediction, the strong performance of GAT-GO [49] indicates that attention-based graph models can exploit structural relations and functional correlations effectively. Similar attention-aware multimodal architectures have also been used to fuse heterogeneous drug, target, and disease information in related biomedical prediction tasks [10,11]. This flexible attention mechanism therefore provides a powerful framework for integrating protein sequence, structure, and ontology information.

### Relation-aware edge attention computation

To better model multi-relation edges in the heterogeneous graph, we introduce a relation-aware edge-attention mechanism. For each edge (*i*,*j*) of relation type *r*, the attention score is computed as


$$\begin{array}{c} e_{ij}^{r}=\gamma_{r}\cdot LeakyReLU\left({𝐚}^{⊤}\left[W{𝐡}_{i}\;\|\;W{𝐡}_{j}\;\|\;{\tilde{𝐫}}_{ij}\right]\right), \end{array}$$ (4)

where ${\tilde{𝐫}}_{ij}$ denotes the learned representation of relation type *r*, $\gamma_{r}$ is a learnable scalar relation weight, and *W* is shared across relation types. This formulation allows different biological relations to influence the attention scores through their relation representations and scalar weights.

### Neighborhood aggregation and node update

The attention scores are normalized over all neighbors of the target node:


$$\begin{array}{c} \alpha_{ij}^{r_{ij}}=\frac{\exp\left(e_{ij}^{r_{ij}}\right)}{\sum_{k\in {𝒩}_{i}}\exp\left(e_{ik}^{r_{ik}}\right)}, \end{array}$$ (5)

where ${𝒩}_{i}$ denotes the complete neighbor set of node *i*. Node features are then updated through weighted aggregation:


$$\begin{array}{c} {𝐡}_{i}^{\prime }=\sigma \left(\sum_{j\in {𝒩}_{i}}\alpha_{ij}^{r_{ij}}W{𝐡}_{j}\right), \end{array}$$ (6)

where *W* is the node transformation shared across relation types and σ(·) is an activation function. Relation types therefore modulate aggregation through the attention weights.

### Multi-head attention fusion

In graph attention networks, multi-head attention [50] is essential for improving representation capacity and training stability. Each GAT layer uses $K_{att}$ independent attention heads in parallel, with each head learning its own attention weights and aggregation patterns. For node *i* at layer *l*, the output of the *k*-th attention head is denoted by ${𝐡}_{i}^{\left(l+1,k\right)}$. The outputs of the attention heads in the two layers contro*l*led by num_layers are concatenated for propagation to the next stage:


$$\begin{array}{c} {𝐡}_{i}^{\left(l+1\right)}={\|}_{k=1}^{K_{att}}{𝐡}_{i}^{\left(l+1,k\right)}. \end{array}$$ (7)

For representation fusion, the multi-head outputs of each attention stage, including the additional attention-based output projection, are averaged and L2-normalized:


$$\begin{array}{c} {\bar{𝐡}}_{i}^{\left(l+1\right)}={\mathrm{Norm}}_{2}\left(\frac{1}{K_{att}}\sum_{k=1}^{K_{att}}{𝐡}_{i}^{\left(l+1,k\right)}\right). \end{array}$$ (8)

Here, ${\mathrm{Norm}}_{2}(\cdot )$ denotes L2 normalization. By leveraging multi-head attention, the model gains stronger generalization and greater robustness on complex biological graph tasks.

### Representation fusion and function prediction

The final decoder representation is formed by concatenating the L2-normalized type-specific input projection, the L2-normalized head-averaged outputs of the two attention layers controlled by num_layers, and the L2-normalized head-averaged output of the additional attention-based projection. With a hidden dimension of 64, this produces a 256-dimensional representation for each protein or GO node. We then formulate protein function prediction as association prediction between protein nodes and GO nodes. Let the representation of protein node *i* be ${𝐡}_{i}$ and that of GO term node *j* be ${𝐠}_{j}$. We use a dot-product decoder to score their compatibility:


$$\begin{array}{c} s_{ij}={𝐡}_{i}^{⊤}{𝐠}_{j}. \end{array}$$ (9)

The score is then mapped to an association probability through the sigmoid function:


$$\begin{array}{c} {\hat{y}}_{ij}=\sigma \left(s_{ij}\right), \end{array}$$ (10)

where ${\hat{y}}_{ij}$ denotes the predicted confidence that protein *i* is annotated with GO term *j*. This decoder is parameter-efficient and computationally lightweight, making it suitable for large-scale candidate protein–GO prediction.

### Graph Decomposition Network (GDN)

After obtaining node representations from the heterogeneous graph attention encoder, we further introduce GDN to factorize node representations. Specifically, GDN splits each node representation along the feature dimension into *M* factor subspaces, where $𝐇=[{𝐇}^{\left(1\right)}\|\cdots \|{𝐇}^{\left(M\right)}]$ and ${𝐇}^{\left(m\right)}\in {\mathbb{R}}^{n\times d/M}$. In our experiments, we use uniform partitioning. This decomposition aims to disentangle the fused representation into several relatively independent and complementary semantic components, mitigating semantic entanglement induced by heterogeneous relations and yielding more discriminative multi-factor representations for function prediction.

### Negative sample selection

To alleviate extreme class imbalance and sharpen decision boundaries, we adopt a two-stage strategy that combines online hard negative mining with random negative completion during training. The sampled pairs are used as training targets rather than as confirmed absent biological functions, and negative sampling follows the reviewed-only supervision scheme. The number of sampled negative pairs is


$$\begin{array}{c} |{𝒩}_{\text{neg}}|=P_{e}\cdot |{𝒩}_{\text{pos}}|, \end{array}$$ (11)

where $\left|{𝒩}_{\text{pos}}\right|$ denotes the number of positive samples and $P_{e}$ is the negative sampling ratio, that is, the number of sampled negatives per positive instance. Specifically, for each reviewed protein *i*, we first mask its positive GO terms in the GO term space, then score each remaining candidate GO term *j* with the current model, and select the highest-scoring candidate terms as hard negatives near the decision boundary. The number of hard negatives is


$$\begin{array}{c} |{𝒩}_{\text{hard}}|=P_{h}\cdot |{𝒩}_{\text{neg}}|, \end{array}$$ (12)

where $P_{h}$ controls the proportion of hard negatives among all sampled negatives. We then uniformly sample additional negatives from the candidate GO space of the same reviewed protein and use a sparse lookup matrix (protein × GO) derived from the training set to filter out positive entries and already selected hard negatives. After filtering and replacement, positive samples are assigned training target 1, sampled negatives are assigned training target 0, and both are concatenated for decoding. The resulting negative annotation edges are likewise restricted to reviewed proteins during training. This procedure continually mines challenging candidate pairs for optimization, but the sampled negatives should not be interpreted as curated biological negatives.

### Loss function optimization

To encourage the *M* factor subspaces learned by GDN to be complementary yet relatively independent, and to suppress redundancy and collapse across factors, we introduce two disentanglement regularizers on top of the main-task loss and combine them with the prediction loss to form the final objective.

### Regularization strategy

To promote effectiveness and independence across factors, we define two regularization terms. **Inter-factor node repulsion** encourages representations of the same node in different factor subspaces to be dissimilar, thereby reducing redundancy:


$$\begin{array}{c} {ℒ}_{\text{inter-f}}=\sum_{i}\sum_{p\neq q}\cos\left({𝐡}_{i}^{\left(p\right)},{𝐡}_{i}^{\left(q\right)}\right). \end{array}$$ (13)

**Inter-factor space separation** requires the mean vectors of different factor subspaces to be separated, thereby enforcing global differences among subspaces:


$$\begin{array}{c} {ℒ}_{\text{inter-s}}=-\sum_{p\neq q}{\left\|{\bar{𝐡}}^{\left(p\right)}-{\bar{𝐡}}^{\left(q\right)}\right\|}_{2}^{2}, \end{array}$$ (14)

where ${\bar{𝐡}}^{\left(p\right)}$ denotes the mean representation of the *p*-th factor subspace. Minimizing these terms encourages orthogonality and independence among subspaces and helps prevent highly overlapping representations.

### Overall loss function

To ensure accurate protein–GO association prediction under class imbalance, we use weighted BCE as the main-task loss:


$$\begin{array}{c} {ℒ}_{\text{BCE}}=-\sum_{\left(i,j\right)}\left(P_{n}y_{ij}\log{\hat{y}}_{ij}+(1-y_{ij})\log(1-{\hat{y}}_{ij})\right), \end{array}$$ (15)

where ${\hat{y}}_{ij}$ denotes the predicted association probability between protein *i* and GO term *j*, $y_{ij}$ is the training target, with 1 for positive samples and 0 for sampled negatives, and $P_{n}$ is the positive-sample weight. The overall optimization objective is then defined as:


$$\begin{array}{c} ℒ={ℒ}_{\text{BCE}}+\lambda \left({ℒ}_{\text{inter-f}}+{ℒ}_{\text{inter-s}}\right), \end{array}$$ (16)

where λ is the shared regularization hyperparameter applied to both disentanglement terms. Minimizing this composite objective allows the model to capture diverse functional signals while reducing redundancy across factors, thereby improving representation quality and discrimination.

### Implementation details

The model was implemented with PyTorch and the Deep Graph Library (DGL) and trained on a single GPU (cuda:0). Unless otherwise specified, we used the Adam optimizer with learning rate $5\times 10^{-4}$, weight decay $2\times 10^{-4}$, batch size 64, dropout 0.5, LeakyReLU slope 0.01, and a maximum of 1,000 epochs. Early stopping was applied based on validation loss with a patience of 100 epochs. The heterogeneous graph attention encoder used num_layers = 2, followed by an additional attention-based output projection, with hidden dimension 64, 4 attention heads per stage, edge-feature dimension 32, a dot-product decoder, residual connections disabled, and residual-attention weight 0. For hard negative mining, the hard-negative proportion was fixed at $P_{h}=0.5$, the candidate GO pool size was 512 for each protein, and the random seed was fixed to 42. Branch-specific settings for $P_{e}$, $P_{n}$, *M*, and λ are reported in the hyperparameter analysis section. Branch-specific graph sizes, parameter counts, and observed runtimes are reported in Table H in S1 Appendix. Peak GPU memory was not recorded in the current training logs. The complete shared implementation and training settings are listed in Table K in S1 Appendix.

## Supporting information

S1 Appendix Supplementary tables for GPCR-GO, including robustness analyses, biological validation, sensitivity analyses, threshold-dependent evaluation, notation, and implementation details. (PDF)
