# Supplementary material for: GPCR-GO: Relation-aware graph learning for predicting Gene Ontology terms of G protein-coupled receptors
Source: PLoS Comput Biol. 2026 Aug 28;22(8):e1014718. doi: 10.1371/journal.pcbi.1014718 (PMC13537695; doi:10.1371/journal.pcbi.1014718)
Supplement: Supplementary file 1 [file pcbi.1014718.s001.pdf]

S1 Appendix

Supplementary tables for GPCR-GO

GPCR-GO: relation-aware graph learning for predicting Gene Ontology terms of G protein-coupled receptors

Anchi Sun, Yongjing Hao, Yijie Ding, Jing Chen, and Hongjie Wu

Supplementary tables

This file contains Tables A–M supporting the analyses, model specification, and reproducibility of GPCR-GO. GO branches are presented consistently as biological process (BP), cellular component (CC), and molecular function (MF).

Table A. Multi-seed performance of GPCR-GO across BP, CC, and MF

Seed	BP			CC			MF		
	Fmax	AUPR	ROC-AUC	Fmax	AUPR	ROC-AUC	Fmax	AUPR	ROC-AUC
42	0.514	0.302	0.862	0.767	0.623	0.938	0.631	0.575	0.947
1	0.534	0.225	0.879	0.789	0.649	0.963	0.677	0.630	0.964
2025	0.524	0.263	0.882	0.783	0.663	0.958	0.664	0.645	0.959
2026	0.517	0.208	0.879	0.791	0.655	0.967	0.638	0.638	0.969
3407	0.572	0.270	0.839	0.801	0.647	0.949	0.686	0.685	0.967
Mean ± SD	0.532 ± 0.024	0.254 ± 0.037	0.868 ± 0.018	0.786 ± 0.013	0.647 ± 0.015	0.955 ± 0.012	0.659 ± 0.024	0.635 ± 0.039	0.961 ± 0.009

Table B. GO annotation enrichment of structural similarity edges compared with PPI and endpoint-matched random controls

Aspect	Edge set	Relation ID	Raw records	Unique pairs	GO-evaluable pairs	Shared GO %	Key GPCR GO %	Mean GO Jaccard
BP	SSE	2	3000	3000	1121	48.2	23.3	0.404
BP	PPI	1	3055	3055	80	35.0	31.3	0.093
BP	Endpoint-matched random	-	-	3000	325	33.5	16.3	0.275
CC	SSE	2	3000	3000	2366	98.2	92.0	0.786
CC	PPI	1	3055	3055	87	93.1	90.8	0.207
CC	Endpoint-matched random	-	-	3000	717	89.5	80.5	0.681
MF	SSE	2	3000	3000	2363	99.0	97.3	0.859
MF	PPI	1	3055	3055	65	41.5	41.5	0.179
MF	Endpoint-matched random	-	-	3000	714	94.5	94.3	0.750

Table C. Recovery of test annotations stratified by GO-term training frequency

GO branch	Training frequency	GO terms with test positives	Test positives	Recovered@10	Recall@10	Term hit rate
BP	0	19	19	0	0.0000	0.0000
	1-5	207	226	75	0.3319	0.3527
	6-20	240	355	110	0.3099	0.3125
	21-100	98	390	231	0.5923	0.3571
	>100	22	370	305	0.8243	0.9091
CC	0	8	8	0	0.0000	0.0000
	1-5	42	44	1	0.0227	0.0238
	6-20	36	48	17	0.3542	0.3611
	21-100	50	193	114	0.5907	0.6600
	>100	12	504	484	0.9603	1.0000
MF	0	2	2	0	0.0000	0.0000
	1-5	25	27	0	0.0000	0.0000
	6-20	79	130	45	0.3462	0.3544
	21-100	49	264	231	0.8750	0.8571
	>100	5	416	415	0.9976	1.0000

Table D. Protein-level diagnostic effect of structural similarity edges and association with PPI degree

Branch	N	mean ΔRecall@10	mean ΔnDCG@10	mean ΔAP	%ΔnDCG@10 > 0	Spearman(PPI degree, ΔnDCG@10)
BP	140	+0.0195	+0.0064	+0.0004	20.7%	r=0.0288, p=0.7358
CC	163	-0.0114	+0.0045	+0.0142	21.5%	r=-0.0069, p=0.9306
MF	155	+0.0001	+0.0141	+0.0157	24.5%	r=0.0501, p=0.5358

Table E. Summary of graph components, input representations, and prediction targets in GPCR-GO

Information source	Graph component	Input representation	Role in model	Output relevance
Protein sequence	Protein node, node_type = 0	Mean-pooled ESM-2 embedding for each protein. The preprocessing script uses esm2_t33_650M_UR50D, with sequences truncated to 1022 residues.	Provides the initial protein feature vector before graph message passing.	Protein representations are used to score candidate GO terms for each protein.
GO terms from UniProt annotations	GO term node, node_type = 1; BP, CC, and MF are processed as separate graphs	One-hot feature vector for each GO term within the corresponding ontology aspect.	Defines the label-node space and provides trainable GO-term representations.	Each GO node is a candidate output label for protein-GO prediction.
Protein-GO annotations	Protein-GO edge from protein node to GO node	Binary positive association with weight 1.0, extracted from UniProt GO columns after filtering to reviewed proteins.	Provides the supervised signal for learning protein-GO associations. Negative protein-GO pairs are sampled during training.	This is the direct prediction target: the model outputs a score/probability for each protein-GO pair.
Protein-protein interaction (PPI)	Protein-protein edge between two protein nodes	Physical interaction edges filtered from BioGRID so both endpoints are GPCR proteins; edge weight is 1.0.	Allows information propagation among interacting proteins and captures functional association beyond sequence alone.	Indirectly improves GO prediction by enriching the learned protein representation.
Structural similarity	Protein-protein edge between structurally similar proteins	Structural-similarity edges are built by selecting the top-K protein pairs with the highest cosine similarity, with K = 3000 to keep the structural graph comparable in scale to the PPI network, which contains 3,055 edges.	Connects proteins with similar structural profiles and complements PPI-based neighborhoods.	Indirectly improves GO prediction by allowing structure-based message passing among proteins.
GO hierarchy	GO-GO hierarchy edge from child GO term to parent GO term	Parent-child relations parsed from the *is_a* and *part_of* entries in the GO OBO file; both are encoded as a single GO-GO hierarchy relation (relation type 3) with edge weight 1.0.	Preserves the ontology organization of GO labels and clarifies dependencies among functional terms.	Supports hierarchy-aware interpretation of predicted GO labels. The final output remains protein-GO association scores.
Model output and supervision	Protein-GO pair scoring after relation-aware graph attention	Node embeddings from relation-aware GAT are decoded by a dot-product decoder. Training uses BCEWithLogitsLoss with sampled negatives and optional hard negative mining.	Learns an embedding space in which true protein-GO pairs receive higher scores than negative pairs.	For each test protein, the model scores all GO terms in the corresponding BP, CC, or MF graph and evaluates the resulting multi-label predictions.

Table F. Configuration summary of GPCR-GO and comparative baseline methods

Method	Model type	Key input / sequence representation	Other biological information	Training setting
Naive	Frequency baseline	GO-term frequencies estimated from the GPCR training set	None	Non-trainable; training-set frequencies were evaluated on the held-out test split.
BLAST	Homology baseline	Raw protein sequences; annotations transferred from similar training proteins	Sequence similarity only	Non-trainable; the training split was used as the reference database.
DeepGOCNN	Sequence CNN	Amino-acid sequence with the original CNN feature pipeline; no ESM2 in the original CNN branch	None	Retrained with the original implementation's recommended input format, epochs, and hyperparameters.
DeepFRI	Structure-aware GCN	Original sequence features plus protein contact/structure graph	Protein contact/structure graph	Retrained with the author-recommended training schedule and model-specific hyperparameters.
DeepGO-SE	Ontology-aware semantic-entailment model	ESM2-based protein representation in the original framework	GO axioms and semantic constraints	Retrained or fine-tuned with the original configuration; the same validation split was used for model selection.
TALE+	Sequence-label embedding model	Transformer-based joint protein sequence and GO-label representation	GO label hierarchy/label embedding and sequence-similarity component	Retrained with author-recommended epochs and hyperparameters.
HEAL	Hierarchical graph transformer	Pretrained protein-language-model residue features, such as ESM-1b in the official implementation	Protein structure graph and contrastive learning	Retrained with the original HEAL input format, training schedule, and tuning strategy.
GALA	Graph transformer with adversarial alignment	Sequence/protein-language-model residue features and structure graph	Structural graph, adversarial domain alignment, and label-embedding alignment	Retrained with the original GALA training protocol and author-recommended hyperparameters.
TAWFN	Adaptive sequence-structure fusion model	Protein sequence, contact map, one-hot features, and deep semantic sequence features	Protein structure/contact map and CNN-GCN adaptive fusion	Retrained with the original TAWFN training schedule and model-specific hyperparameters.
GPCR-GO-Sup	Supervised GPCR-GO variant	ESM2 embeddings for reviewed GPCR proteins	GO hierarchy, PPI edges, and structural-similarity edges; no unlabeled GPCR nodes	Trained with GPCR-GO settings; validation split used for early stopping and model selection.
GPCR-GO	Semi-supervised heterogeneous graph model	esm2 embeddings for reviewed and unreviewed GPCR proteins	GO hierarchy, PPI edges, structural-similarity edges, reviewed annotation edges, and unlabeled GPCR nodes	Trained with GPCR-GO settings; validation split used for early stopping and branch-specific hyperparameter selection.

Table G. Sensitivity analysis of the hard-negative proportion P_h

Aspect	P_h requested	P_h effective	P_e	P_n	Fmax	AUPR	Smin
BP	0.000	0.000	15	15	0.368	0.124	16.780
BP	0.250	0.200	15	15	0.522	0.281	15.641
BP	0.500	0.467	15	15	0.514	0.302	16.374
BP	0.750	0.733	15	15	0.445	0.190	16.139
BP	1.000	1.000	15	15	0.481	0.193	16.187
CC	0.000	0.000	15	15	0.642	0.471	3.589
CC	0.250	0.200	15	15	0.759	0.573	3.586
CC	0.500	0.467	15	15	0.748	0.594	3.683
CC	0.750	0.733	15	15	0.763	0.509	3.710
CC	1.000	1.000	15	15	0.747	0.485	3.734
MF	0.000	0.000	15	15	0.509	0.510	4.861
MF	0.250	0.200	15	15	0.640	0.602	4.210
MF	0.500	0.467	15	15	0.631	0.575	4.572
MF	0.750	0.733	15	15	0.525	0.427	4.983
MF	1.000	1.000	15	15	0.542	0.465	4.832

Table H. Graph and split statistics of the branch-specific GPCR-GO datasets, with model size and observed runtime

**Branch**	**BP**	**CC**	**MF**
Total nodes	30,483	28,051	28,280
Protein nodes	27,677	27,677	27,677
Reviewed protein nodes	3,465	3,465	3,465
Unreviewed protein nodes	24,212	24,212	24,212
GO nodes	2,806	374	603
Training protein–GO edges	19,304	8,164	7,251
Validation protein–GO edges	1,425	760	806
Test protein–GO edges	1,360	797	839
PPI records in `link.dat`	3,055	3,055	3,055
Structural-similarity edges	3,000	3,000	3,000
GO hierarchy edges	781	166	236
Total records in `link.dat`	26,140	14,385	13,542
Trainable parameters	443,195	287,547	302,203
Observed training time (seed 42)	497.8 min	136.4 min	111.7 min

Note: Node and edge counts were obtained from the branch-specific graph files used in this study. The `link.dat` totals comprise training protein–GO annotations, PPI records, structural-similarity edges, and GO hierarchy edges. Parameter counts were obtained from the current branch-specific checkpoints. Training time is the wall-clock interval between the start and end records in the seed-42 training log; the current logs did not record peak GPU memory.

Table I. Threshold-dependent test performance using validation-selected thresholds

**Metric**	**BP**	**CC**	**MF**
Validation-selected threshold	0.65	0.42	0.88
Precision	0.3065	0.4286	0.8548
Recall	0.6359	0.8762	0.3043
F1	0.4030	0.5727	0.4488
micro-F1	0.2869	0.2560	0.4408
macro-F1	0.1387	0.1514	0.0157
Fmax	0.5142	0.7668	0.6314
micro-AUPR	0.3018	0.6233	0.5751
macro-AUPR	0.2993	0.2925	0.4168

Table J. Unified symbols, abbreviations, notation, and model dimensions used in GPCR-GO

**Symbol / abbreviation**	**Type**	**Meaning**	**Dimension / value**
GPCR-GO	Model name	Proposed GPCR-specific Gene Ontology function-prediction framework.	–
GO	Abbreviation	Gene Ontology.	–
BP	GO branch	Biological process; reported first in branch-specific results.	–
CC	GO branch	Cellular component; reported second in branch-specific results.	–
MF	GO branch	Molecular function; reported third in branch-specific results.	–
PPI	Abbreviation	Protein-protein interaction.	–
SSE	Abbreviation	Structural-similarity edge.	–
SSL	Abbreviation	Semi-supervised learning.	–
GDN	Abbreviation	Graph Decomposition Network.	–
HNM	Abbreviation	Hard negative mining.	–
DSSP	Structural-feature tool	Tool used to extract secondary-structure and solvent-accessibility descriptors.	–

Continued on next page

Table J continued

Symbol / abbreviation	Type	Meaning	Dimension / value
pLDDT	Structure-confidence metric	AlphaFold confidence score used to filter predicted structures.	Scalar per residue
ϕ, ψ	Structural symbols	Backbone dihedral angles used in DSSP-derived residue descriptors.	Scalars per residue
RSA	Structural descriptor	Relative solvent accessibility.	Scalar per residue
$\mathrm{IC}(t)$	Information-content metric	Term-level information content of GO term t in the final GO label set.	Scalar
$\mathrm{IC}_{\mathrm{ann}}(t)$	Information-content metric	Annotation-level information content of GO term t, treating each protein-term pair as one annotation instance.	Scalar
$\Delta\mathrm{IC}$	Information-content summary	Difference between median term-level IC and median annotation-level IC.	Scalar
N_{prot}	Dataset-count symbol	Number of annotated proteins used when calculating term-level IC.	3,465
$n_{\mathrm{prot}}(t)$	Dataset-count symbol	Number of proteins annotated with GO term t in the final GO label set.	Scalar count
N_{ann}	Annotation-count symbol	Total number of protein-term annotation instances.	Branch dependent
$n_{\mathrm{ann}}(t)$	Annotation-count symbol	Number of annotation instances assigned to GO term t.	Scalar count
$\mathbf{z}_i$	Structural representation	Protein-level structural vector for protein i obtained by mean/std pooling of DSSP-derived residue descriptors.	26
S_{ij}	Structural-similarity score	Cosine similarity between the structural vectors of proteins i and j.	Scalar
K_{SSE}	Graph-construction hyperparameter	Number of top structural protein pairs retained as structural-similarity edges.	3,000
E_{struct}	Graph notation	Set of structural-similarity edges in the heterogeneous graph.	Variable-size edge set
N_{p}	Graph-size symbol	Number of protein nodes, including 3,465 reviewed and 24,212 unreviewed proteins.	27,677
N_{GO}	Graph-size symbol	Number of GO nodes in the branch-specific graph.	BP: 2,806; CC: 374; MF: 603
X_{p}	Input feature	Initial protein feature from ESM2.	1,280
X_{g}	Input feature	Initial one-hot feature for GO nodes.	N_{GO}
P_{protein}	Input projection	Protein input projection.	$1{,}280 \times 64$
P_{GO}	Input projection	GO input projection.	$N_{\mathrm{GO}} \times 64$
d_{h}	Model dimension	Hidden dimension per attention head.	64
K_{att}	Model hyperparameter	Number of independent attention heads in a graph attention layer.	4
L	Model hyperparameter	Number of hidden graph-attention layers.	2
R	Relation-count symbol	Number of original relation types in `link.dat`.	4
R'	Relation-count symbol	Expanded edge-type vocabulary including reverse edges and self-loops.	$2R + 1 = 9$
q_r	Relation feature	One-hot vector for edge type r.	9
B_{edge}	Relation embedding	Learnable edge-type embedding table.	9×32
e_r	Relation embedding	Edge-type embedding for relation r.	32
ϕ_e	Edge-feature transform	MLP applied to edge-type embeddings.	$32 \to 64 \to 128$, reshaped as 4×32
γ_r	Relation parameter	Learnable scalar relation weight for relation type r.	9 scalars
W_0	Node projection	First graph-attention node projection.	64×256
W_1	Node projection	Second graph-attention node projection.	256×256
W_{out}	Node projection	Output graph-attention node projection.	256×256
a_l, a_r	Attention parameters	Left and right attention vectors.	Each $1 \times 4 \times 64$

Continued on next page

Table J continued

Symbol / abbreviation	Type	Meaning	Dimension / value
a_e	Attention parameter	Edge attention vector.	$1 \times 4 \times 32$
r	Relation-type index	Edge-relation type in the heterogeneous graph.	Scalar index
α_{ij}^r	Attention coefficient	Normalized attention weight from node j to node i under relation type r.	Scalar
$\mathbf{h}_i, \mathbf{g}_j$	Decoder representations	Final protein-node and GO-node representations used by the dot-product decoder.	256 each
s_{ij}	Decoder score	Compatibility score $s_{ij} = \mathbf{h}_i^\top \mathbf{g}_j$.	Scalar
P_e	Training hyperparameter	Negative sampling ratio; number of sampled negative instances per positive instance for reviewed proteins.	Branch specific; see Table L
P_n	Training hyperparameter	Positive-sample weight in the weighted binary cross-entropy loss.	Branch specific; see Table L
P_h	Training hyperparameter	Hard-negative proportion among all sampled negative instances.	0.5
M	GDN hyperparameter	Number of factor subspaces in the Graph Decomposition Network.	Branch specific; see Table L
λ	Regularization hyperparameter	Shared weight applied to the inter-factor node-repulsion and inter-factor space-separation terms.	Branch specific; see Table L
λ_1, λ_2	Regularization notation	Component regularization weights tied as $\lambda_1 = \lambda_2 = \lambda$.	Scalars
$\mathcal{L}_{\text{BCE}}$	Loss term	Weighted binary cross-entropy loss for protein-GO association prediction.	Scalar
$\mathcal{L}_{\text{inter-f}}$	Loss term	Inter-factor node-repulsion regularizer.	Scalar
$\mathcal{L}_{\text{inter-s}}$	Loss term	Inter-factor space-separation regularizer.	Scalar
y_{ij}	Label symbol	Training target for protein-GO pair (i, j): 1 for an annotated positive and 0 for a sampled pseudo-negative.	Binary scalar
$\hat{y}_{ij}$	Prediction symbol	Predicted association probability between protein i and GO term j.	Scalar in $[0, 1]$

Table K. General implementation and training hyperparameters used for GPCR-GO

Parameter	Value	Notes
Implementation framework	PyTorch and DGL	As reported in the manuscript implementation details.
Training device	Single NVIDIA GeForce RTX 3090 GPU (cuda:0)	Used for all GPCR-GO training runs.
Optimizer	Adam	Applied unless otherwise specified.
Learning rate	5×10^{-4}	Shared training setting.
Weight decay	2×10^{-4}	Shared training setting.
Batch size	64	Shared training setting.
Dropout	0.5	Shared training setting.
LeakyReLU slope	0.01	Used in the heterogeneous graph attention encoder.
Maximum epochs	1,000	Training upper bound.
Early stopping	Validation loss, patience = 100 epochs	Validation split used for model selection and early stopping.
Encoder layers	2	Heterogeneous graph attention encoder.
Hidden dimension	64	Heterogeneous graph attention encoder.
Attention heads per layer (K_{att})	4	Heterogeneous graph attention encoder.
Edge-feature dimension	32	Heterogeneous graph attention encoder.
Decoder	Dot-product decoder	Scores protein-GO candidate pairs.
Residual connections	Disabled	As reported in implementation details.
Residual-attention weight	0	As reported in implementation details.
Hard-negative proportion (P_h)	0.5	Fixed during hyperparameter analysis of P_e and P_n.
Candidate GO pool size	512 per protein	Used for hard-negative mining.
Random seed	42	Fixed for reproducibility.
Structural edge count (K_{SSE})	3,000	Top structural-similarity pairs retained to obtain an edge count comparable to the 3,055 PPI edges.

Table L. Branch-specific hyperparameter settings evaluated on the held-out test split

GO branch	P_e	P_n	P_h	M	λ	Evaluation note
BP	15	15	0.5	8	0.1	P_e/P_n and the GDN setting were evaluated in test-set sensitivity analyses.
CC	10	15	0.5	2	0.1	P_e/P_n and the GDN setting were evaluated in test-set sensitivity analyses.
MF	15	15	0.5	4	1.0	P_e/P_n and the GDN setting were evaluated in test-set sensitivity analyses.
